# Curcumin Pharmacokinetic and Pharmacodynamic Evidences in Streptozotocin-Diabetic Rats Support the Antidiabetic Activity to Be via Metabolite(s)

**DOI:** 10.1155/2015/678218

**Published:** 2015-05-03

**Authors:** Vânia Ortega Gutierres, Michel Leandro Campos, Carlos Alberto Arcaro, Renata Pires Assis, Helen Mariana Baldan-Cimatti, Rosângela Gonçalves Peccinini, Silvia Paula-Gomes, Isis Carmo Kettelhut, Amanda Martins Baviera, Iguatemy Lourenço Brunetti

**Affiliations:** ^1^Department of Clinical Analysis, School of Pharmaceutical Sciences, São Paulo State University (UNESP), Rodovia Araraquara-Jaú Km 01, 14801-902 Araraquara, SP, Brazil; ^2^Department of Natural Active Principles and Toxicology, School of Pharmaceutical Sciences, São Paulo State University (UNESP), Rodovia Araraquara-Jaú Km 01, 14801-902 Araraquara, SP, Brazil; ^3^Department of Biochemistry and Immunology, School of Medicine, University of São Paulo (USP), Avenida Bandeirantes 3900, 14040-900 Ribeirão Preto, SP, Brazil

## Abstract

This study measures the curcumin concentration in rat plasma by liquid chromatography and investigates the changes in the glucose tolerance and insulin sensitivity of streptozotocin-diabetic rats treated with curcumin-enriched yoghurt. The analytical method for curcumin detection was linear from 10 to 500 ng/mL. The *C*_max⁡_ and the time to reach *C*_max⁡_ (*t*_max⁡_) of curcumin in plasma were 3.14 ± 0.9 *μ*g/mL and 5 minutes (10 mg/kg, i.v.) and 0.06 ± 0.01 *μ*g/mL and 14 minutes (500 mg/kg, p.o.). The elimination half-time was 8.64 ± 2.31 (i.v.) and 32.70 ± 12.92 (p.o.) minutes. The oral bioavailability was about 0.47%. Changes in the glucose tolerance and insulin sensitivity were investigated in four groups: normal and diabetic rats treated with yoghurt (NYOG and DYOG, resp.) and treated with 90 mg/kg/day curcumin incorporated in yoghurt (NC90 and DC90, resp.). After 15 days of treatment, the glucose tolerance and the insulin sensitivity were significantly improved in DC90 rats in comparison with DYOG, which can be associated with an increase in the AKT phosphorylation levels and GLUT4 translocation in skeletal muscles. These findings can explain, at least in part, the benefits of curcumin-enriched yoghurt to diabetes and substantiate evidences for the curcumin metabolite(s) as being responsible for the antidiabetic activity.

## 1. Introduction

Curcumin (diferuloylmethane) is a yellow pigment isolated from the dried rhizomes of* Curcuma longa* L. (turmeric). In Asian countries, turmeric is largely used as a dietary spice. Furthermore, traditional Indian and Chinese medicines have used turmeric for the treatment of a diversity of diseases [1]; curcumin has been cited as the main phytochemical responsible for the turmeric beneficial effects. Crescent attention has been given to understand the role of curcumin in the prevention and treatment of various chronic diseases, such as cancer [2, 3], cardiovascular diseases [4], and metabolic disturbances, including obesity [5] and diabetes mellitus [6].

A wide range of biological activities has been attributed to curcumin; however the translation of its experimental biological benefits into clinical trials is difficult due to the low bioavailability of this pigment when administered orally, observed in both rodents [7] and humans [8], which is explained by its poor absorption due to the low solubility in water, limited tissue distribution, and rapid rate of metabolism in liver and intestine followed by the rapid excretion from the body [9]. So the low bioavailability of curcumin appears as a major barrier to reach its adequate circulating levels related to desirable pharmacodynamic actions, hindering its clinical approval as a therapeutic agent for several diseases.

Attempting to overcome this situation, several vehicles and associations with curcumin have been tested, such as curcumin-loaded nanoparticles [10], complexing with phospholipids [2], microemulsifying [11], and association with drug bioenhancers, for example, piperine [12], among others [13]. Previous study from our laboratory [14] showed that curcumin incorporated in yoghurt improved several physiological and biochemical parameters of streptozotocin- (STZ-) diabetic rats treated for 31 days: the food and water intake and the urinary volume were decreased and the body weight gain was increased; the plasma levels of glucose, triglycerides, and transaminases and the urinary levels of glucose, urea, and protein in urine were decreased, when compared with nontreated diabetic rats. These biomarkers were not changed in nondiabetic rats treated with curcumin-enriched yoghurt. With the objective to increase the antidiabetic activity of curcumin, our laboratory recently investigated its coadministration with piperine, which inhibits the biotransformation processes occurring in liver and intestine, increasing the bioavailability of drugs; notably, the treatment of STZ-diabetic rats with both curcumin and piperine in yoghurt for 45 days did not change or even nullified, in the dose-dependent way, the antidiabetic and antioxidant activities of curcumin [15], suggesting that the biotransformation is important to these pharmacological actions of curcumin. In light of these data, the investigation of the pharmacokinetic profile of curcumin when administered with yoghurt in diabetic animals became interesting which, as far as we know, has not yet been described.

The aim of this study was to evaluate the pharmacokinetics of curcumin in diabetic rats when administered intravenously and orally (incorporated in yoghurt) and also to investigate the changes in the glucose tolerance and insulin sensitivity of diabetic rats after acute (1 day) and subchronic (15 days) treatments with curcumin incorporated in yoghurt.

## 2. Material and Methods

### 2.1. Chemical and Reagents

Curcumin (≥77%, 28260, Fluka-Sigma Aldrich, St. Louis, MO, USA) and analytical standard curcumin (≥98.0%, 08511, Fluka-Sigma Aldrich, St. Louis, MO, USA), streptozotocin (STZ, Sigma Aldrich, St. Louis, MO, USA), dinitrophenol, and dimethyl sulfoxide (DMSO) were obtained from Sigma Aldrich (St. Louis, MO, USA); ketamine and xylazine were obtained from Agener União (Embu-Guaçu, SP, Brazil); acetonitrile and methanol (HPLC grade) were obtained from J. T. Baker (Mexico City, Mexico); acetic acid was obtained from Qhemis (Indaiatuba, SP, Brazil); and insulin (Biohulin NU-100; 100 units/mL) was obtained from Biobras (Montes Claros, MG, Brazil). Sodium hydroxide was purchased from Cetus (Santo Amaro, SP, Brazil) and plain yoghurt from Nestlé (Araras, SP, Brazil). Water was purified by a Millipore system (EMD Millipore, Darmstadt, Germany).

### 2.2. HPLC Analysis of Curcumin

The high performance liquid chromatography (HPLC) system consisted of a Waters 600E with a 50 *μ*L sample loop coupled to UV-Vis detector (Waters 2487) set to 420 nm and a computer system for data acquisition (Empower software, Waters) was used. The separation was achieved using a reversed phase Symmetry C_18_ column (4.6 × 250 mm, particle size 5 *μ*m). Mobile phase consisting of methanol, acetonitrile, and 5% acetic acid (35 : 50 : 15, v/v) was employed at a flow rate of 1.0 mL/min.

### 2.3. Preparation of the Standard Solutions and Quality Control of Samples

Standard solutions of curcumin (≥98%, 08511, Fluka-Sigma Aldrich, St. Louis, MO, USA) were prepared in methanol at six concentration levels (0.10, 0.25, 0.75, 1.25, 2.5, and 5 *μ*g/mL). These solutions were diluted in plasma to reach the final plasma concentrations of 10, 25, 75, 125, 250, and 500 ng/mL.

### 2.4. Sample Preparation

An aliquot of 500 *μ*L of rat plasma (blank) or 500 *μ*L of rat plasma containing curcumin (concentrations as described in Section 2.3) were mixed with 10 *μ*L of 0.01 M NaOH and 40 *μ*L of acetonitrile containing 200 *μ*g/mL dinitrophenol; dinitrophenol was used as internal standard (IS). The mixture was vortexed (30 seconds) and 1000 *μ*L of ethyl acetate was added and vortexed again, followed by centrifugation at 1000 ×g for 15 minutes at 10°C. After that, 500 *μ*L of supernatant was transferred to a new clean plastic microtube and evaporated under vacuum for 15 minutes at 37°C in a Mini Vac Sample Concentrator Range-Genevac. Lastly, the dry sample was reconstituted by 125 *μ*L of methanol and injected into the HPLC system.

### 2.5. Method Validation

In order to determine the confidence limits of the bioanalytical method, the validation was performed based on the RDC-27/2012 from Agência Nacional de Vigilância Sanitária (ANVISA, Brazil) and the* Guidance for Industry: Bioanalytical Method Validation 2001* from* Food and Drug Administration* (FDA, USA). The analytical methodology was validated regarding selectivity, linearity, accuracy, precision, limit of detection (LOD), lower limit of quantitation (LLOQ), recovery, and stability.

The selectivity evaluation of the method was performed by the analysis of a blank plasma sample in order to determine the presence of interfering peaks in curcumin and IS retention times.

Linearity was achieved by plotting the curcumin/IS ratio versus nominal concentrations. The acceptance criteria for linearity were accuracy as percentage of the nominal concentration (%) between 85 and 115%, except at the LLOQ, where it can be between 80 and 120% and linear regression coefficient (*r*) higher than 0.98.

Precision and accuracy were evaluated together at four concentration levels or quality controls (QC): high-QC (HQC) 400 ng/mL, medium-QC (MQC) 125 ng/mL, lower-QC (LQC) 25 ng/mL, and the LLOQ-QC 10 ng/mL. The assay was carried out in the same day (intraday, *n* = 5) and in three different days (interday, *n* = 15). The acceptance criterion for precision was a coefficient of variation (%) less than 15%, except at the LLOQ where it can be less than 20%. The accuracy acceptance criterion was a percentage of the nominal concentration (%) between 85 and 115%, except at the LLOQ where it can be between 80 and 120%.

The recovery test, which determines the extraction procedure efficiency, was achieved comparing extracted samples to nonextracted samples (100%) of curcumin in HQC and LQC.

In order to determine whether the sample can be stored in specific conditions, the stability studies were performed. Plasma aliquots were prepared at concentrations of 25 and 400 ng/mL in triplicate and analyzed according to the following conditions:* bench-top stability*, where the samples stay at room temperature (30°C) for 4 h;* postprocessing stability*, where samples stay at room temperature (30°C) for 4 h after extraction;* freeze-thaw stability*, where samples were frozen at −20°C and thawed at room temperature over three cycles during 72 hours;* long-term stability*, where samples were frozen at −20°C for seven days. After these conditions, samples were analyzed and compared with the results obtained from freshly prepared and analyzed samples.

### 2.6. Animals

Male Wistar rats (*Rattus norvegicus*) weighing 140–160 g (6 weeks) were maintained under environmentally controlled conditions of temperature (23 ± 1°C) and humidity (55 ± 5%) and with a 12 h light/dark cycle, having free access to water and normal lab chow diet (Purina Evialis do Brasil Nutrição Animal Ltda., SP, Brazil). Before the beginning of the experiments, the animals were maintained under these conditions for at least 5 days. The experiments were conducted during the light phase and the experimental protocol was approved by the Committee for Ethics in Animal Experimentation of the School of Pharmaceutical Sciences, UNESP, Araraquara (resolution number 37/2012).

### 2.7. Induction of the Experimental Diabetes Mellitus

Experimental diabetes mellitus was induced by a single intravenous injection of STZ (40 mg/kg b.w.) dissolved in 0.01 M citrate buffer (pH 4.5), in previously 14 h fasted rats. Normal rats received only citrate buffer. Four days after STZ administration, diabetic rats with postprandial glycemia values of approximately 400 mg/dL were used in the experiments. Plasma glucose levels were determined by the glucose oxidase method [16] using commercial kit (Labtest Diagnostica SA, Brazil).

### 2.8. Preparation of Curcumin-Enriched Yoghurt

Curcumin (≥77%, 28260, Fluka-Sigma Aldrich, St. Louis, MO, USA) was mixed with 0.5 mL of plain yoghurt, in the doses of 45 or 500 mg/kg b.w., with a homogenizer (Metabo, Marconi, Piracicaba, SP, Brazil) operating at 27,000 rpm for 90 seconds at a controlled temperature of 25°C.

### 2.9. Pharmacokinetics (PK) of Curcumin in Diabetic Rats

Twenty-five diabetic rats (weighing 150–200 g) were divided into two groups: intravenous (i.v.) group (*n* = 10), of which animals were treated with 10 mg/kg of curcumin (16 mg/mL in DMSO), and the oral group (*n* = 15), of which animals were treated with 500 mg/kg of curcumin (single dose) incorporated in 0.5 mL of yoghurt.

Twenty-four hours before the curcumin administration, the animals were submitted to vein and/or artery catheter implantation. These catheters were used to drug administration or to serial blood collections [17]. Eight hours before the curcumin administration, the animals were deprived of food. The sampling times were 5, 10, 20, 30, 45, and 60 minutes after the oral administration and 2.5, 5, 7.5, 10, 20, 30, and 45 minutes after the i.v. administration. Both administrations were performed over a short period of time.

The curcumin PK parameters were calculated based on the concentration in plasma versus time curves. The half-life of elimination (*t*_1/2_) was calculated by the graphic method and the half-life of absorption was calculated by the residues method. The constants of absorption (*k*_*a*_) and elimination (*k*_el_) were calculated by the equation 0.693/*t*_1/2_, where *t*_1/2_ is the half-life of absorption or elimination, respectively. The area under the curve (AUC) of the plasma concentration versus time profile from time zero to the last sampling time (AUC_0-*t*_) was calculated by the trapezoidal rule. The area under the curve from time zero extrapolated to infinity (AUC_0-∞_) was calculated by the equation AUC_0-∞_ = AUC_0-*t*_ + *C*_pn_/*k*_el_ (where *C*_pn_ is the last plasma concentration determined and *k*_el_ is the elimination constant). The AUC_0-∞_ was used for the calculation of the total clearance (Cl = dose/AUC_0-∞_), and the distribution volume (*V*_*d*_) was calculated using the equation *V*_*d*_ = Cl/*k*_el_. The mean residence time (MRT) was estimated from AUMC/AUC, where AUMC is area under the first moment curve. The oral bioavailability (*F*) of curcumin was calculated by the relation between the AUC obtained in the oral administration and the AUC obtained in the intravenous administration. The maximum plasma concentration (*C*_max⁡_) was obtained from the experimental data as well as the time of the occurrence of *C*_max⁡_ (*t*_max⁡_).

### 2.10. Pharmacodynamics (PD) of Curcumin Administered with Yoghurt

#### 2.10.1. Treatment of Animals

Forty rats were divided into four groups: normal rats treated with yoghurt (NYOG) or with 45 mg/kg curcumin-enriched yoghurt twice a day (NC90); diabetic rats treated with yoghurt (DYOG) or with 45 mg/kg curcumin-enriched yoghurt twice a day (DC90). Normal and diabetic rats were treated by gavage at 08:00 h and 17:00 h, for 15 days. Curcumin incorporated in yoghurt (as described in Section 2.8) was administered as a half dose in 0.5 mL, totaling 1.0 mL yoghurt/rat/day of treatment. Control rats received only yoghurt. Glucose and insulin tolerance tests were performed after 1 and 15 days of treatment. Changes in the insulin signaling pathway (total protein content and phosphorylation levels in serine-473 residue of AKT or protein kinase B) and in the content of glucose transporter type 4 (GLUT4) in plasma membrane were also investigated in* gastrocnemius* muscles from NYOG, NC90, DYOG, and DC90 rats treated for 15 days and submitted to a glucose oral overload (as described in Section 2.10.2).

#### 2.10.2. Oral Glucose Tolerance Test (OGTT)

OGTT was performed in 14 h fasted rats. A glucose solution (2.5 g/kg b.w.) was administered orally, and blood samples were collected from the tip of the tail before (*t* = 0) and 15, 30, 45, 60, 75, 90, 105, and 120 minutes after the glucose loading. Results were expressed as mg/dL and the area under the curve (AUC, g/dL/120 min) was calculated.

#### 2.10.3. Insulin Tolerance Test (ITT)

For ITT, 6 h fasted rats received a single intraperitoneal injection of human recombinant insulin (1.5 U/kg b.w.), and blood samples were collected before (*t* = 0) and 5, 10, 15, 20, 25, and 30 minutes after the insulin administration for the plasma glucose measurement. Results were expressed as mg/dL. For the estimation of the insulin sensitivity, the constant rate for glucose disappearance (*k*_itt_, %/min) was calculated using the equation *k*_itt_ = 0.693/*t*_1/2_, where *t*_1/2_ represents the half-life of plasma glucose concentration calculated from the slope of least squares analysis in the interval of time in which glycemia declines linearly after the insulin administration [18].

#### 2.10.4. Western Blotting Analysis

Concerning sample preparation for AKT protein content and phosphorylation levels,* gastrocnemius* muscles were homogenized in 50 mM Tris-HCl buffer (pH 7.4) containing 150 mM NaCl, 1 mM ethylenediaminetetraacetic acid (EDTA), 1% Triton X-100, 1% sodium deoxycholate, 1% SDS, 10 mM sodium pyrophosphate, 100 mM sodium fluoride, 10 mM sodium orthovanadate, 5 *μ*g/mL aprotinin, 1 *μ*g/mL leupeptin, and 1 mM phenylmethylsulfonyl fluoride (PMSF). The homogenates were centrifuged at 15,500 ×g at 4°C for 40 minutes and the supernatant was used for analysis. Protein levels were determined using bovine serum albumin as standard [19].

Concerning sample preparation for GLUT4 content in plasma membrane,* gastrocnemius* muscles were homogenized in 50 mM Tris-HCl buffer (pH 8.0) containing 0.1% NP-40, 0.5 mM dithiothreitol (DTT), and the same proteases and phosphatases inhibitors used in the above buffer. The homogenates were centrifuged at 1,000 ×g at 4°C for 10 minutes. Precipitates were washed twice with the same buffer above described without NP-40 and centrifuged at the same conditions. Precipitates were added in buffer containing 1% NP-40 and then centrifuged at 15,500 ×g at 4°C for 20 minutes; the supernatants were considered the plasma membrane fraction [20].

Supernatants (for AKT and GLUT4 studies) were prepared with sample buffer (125 mM Tris-HCl, 20% glycerol, 4% SDS, 100 mM DTT, 0.02% bromophenol blue, pH 6.8); equal amounts of protein (20 *μ*g) were subjected to SDS-PAGE analysis on 8% acrylamide gels [21] and electroblotted onto nitrocellulose membranes [22].

After blocking, proteins were detected by overnight incubation at 4°C with specific primary antibodies: anti-AKT (1 : 500, Cell Signaling, Danvers, MA, USA), anti-phospho-[Ser-473]-AKT (1 : 500, Cell Signaling, Danvers, MA, USA), and anti-GLUT4 (1 : 1,000, Santa Cruz Biotechnology, Santa Cruz, CA, USA). Anti-*β*-actin (1 : 1,000, Santa Cruz Biotechnology, Santa Cruz, CA, USA) was used as internal control. Primary antibodies were detected by peroxidase-conjugated secondary antibodies (Cell Signaling, Danvers, MA, USA) and visualized with enhanced chemiluminescence reagents (SuperSignal West Pico Chemiluminescent Substrate, Thermo Scientific, Waltham, MA, USA) and detected with C-Digit Blot Scanner (LI-COR, Lincoln, NE, USA). Band intensities were read and analyzed with the LI-COR Image Studio 4.0 Program.

### 2.11. Statistical Analysis

Data were expressed as mean ± standard error of mean (SEM). One-way analysis of variance (ANOVA) followed by Student-Newman-Keuls test was used to compare the intergroup differences and of the AUC. Differences were considered significant at *P* < 0.05. Statistical analyses were performed using the program Graphpad Instat 3.05 (GraphPad Software, USA).

## 3. Results

### 3.1. Method Validation

**Figure 1:**
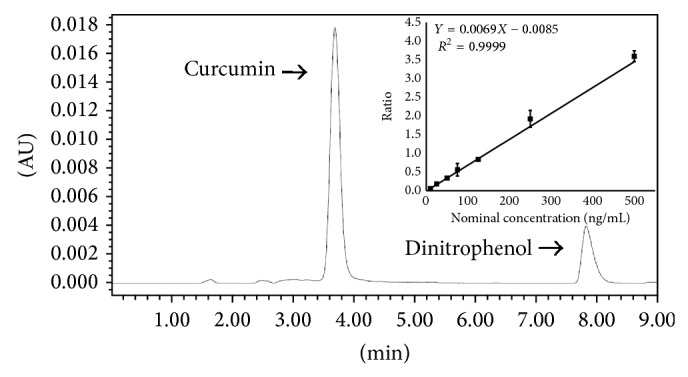
Chromatogram of plasma analysis containing 25 ng/mL curcumin and IS (5.3 *μ*g/mL dinitrophenol) at 420 nm and the linearity of the bioanalytical method.

As observed in the chromatogram (Figure 1), curcumin and IS showed retention times of 4 and 8 minutes, respectively, which shows plasma containing curcumin (25 ng/mL) and IS (5.3 ng/mL) at 420 nm. The selectivity assay showed no interfering peaks of the biological matrix. This method was validated using four quality control levels of the calibration curve. The obtained linearity equation was *y* = 0.0069 − 0.0085*x* and linear regression coefficient was *r* = 0.9999. Each curve concentration level matched to the accuracy acceptance criteria, of which accuracy range was 87.88–105.82%. All precision and accuracy results are within the acceptance criteria (Table 1). The intraday and interday precisions were no more than 13.52% and 12.46%, respectively, while the intraday and interday accuracies were between 86.05 and 113.59% and between 89.65 and 98.74%, respectively. The obtained recovery for curcumin was 67 ± 9.78%; however, there is no acceptance criterion to recovery, since the recovery reproducibility is good enough to stay within the accuracy and precision acceptance criteria. The stability study indicated that curcumin is stable at the evaluated conditions, considering that it has not failed in any of the tests (Table 2).

### 3.2. PK Profile of Curcumin

**Figure 2:**
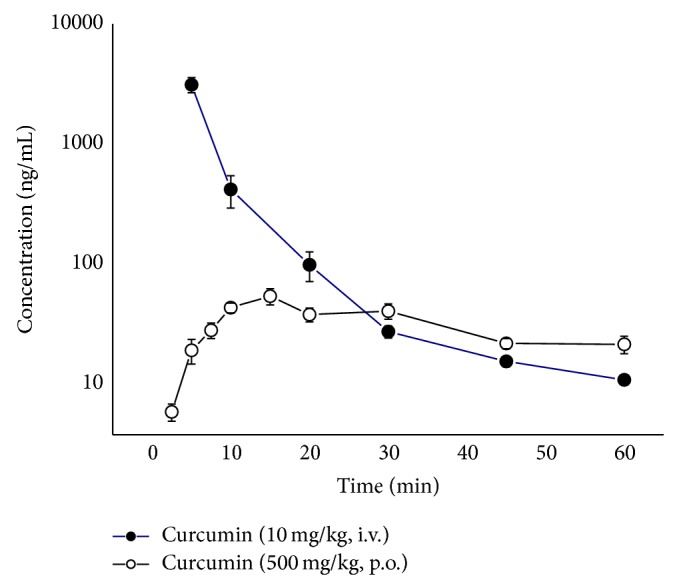
Plasma curcumin concentrations versus time curves in STZ-diabetic rats. Values are expressed as means ± SEM.

PK profile was evaluated after a single administration of 10 mg/kg b.w. of curcumin in DMSO (i.v.) and 500 mg/kg b.w. of curcumin incorporated in yoghurt (p.o.). Curcumin plasma concentration versus time profiles can be seen in Figure 2. Concentration values were best-fitted by a one-compartmental model. Maximum plasma curcumin concentration occurred in about 14 minutes after the oral administration. The intravenously administered curcumin showed extrapolated *C*_max⁡_ of 3.14 ± 0.90 *μ*g/mL, whereas oral administration with a dose 50-fold higher showed 0.06 ± 0.01 *μ*g/mL (Table 3).

The areas under curve (AUC_0-*t*_) were 12.27 ± 2.75 *μ*g/mL/min for intravenous administration and 1.89 ± 0.25 *μ*g/mL/min for oral administration, of which doses were 10 mg/kg and 500 mg/kg, respectively (Table 4).

Although the Cl was not different between these administration routes, *V*_*d*_ for oral administration was higher than *V*_*d*_ for i.v. administration, leading to an increase in the curcumin half-life through oral route (Table 4).

### 3.3. Glucose Tolerance and Insulin Sensitivity after Short- and Long-Term Treatment of Diabetic Rats with Curcumin

**Figure 3:**
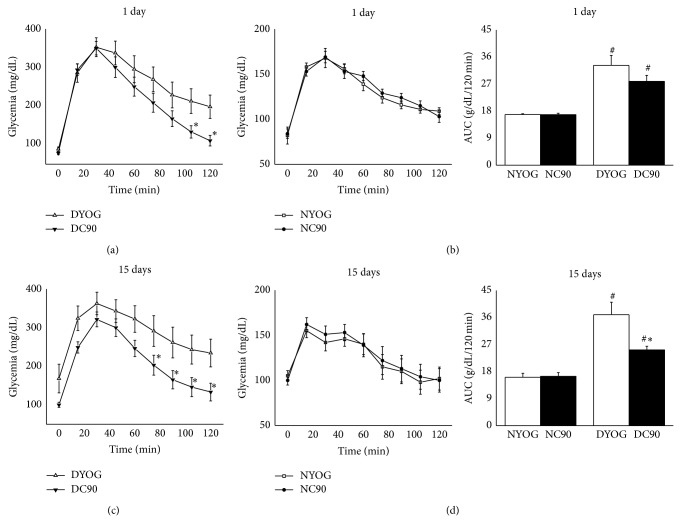
Oral glucose tolerance of normal and STZ-diabetic rats treated with curcumin incorporated in yoghurt. Glycemia levels before (*t* = 0) and after glucose overload in rats treated for 1 day ((a) diabetic; (b) normal) and for 15 days ((c) diabetic; (d) normal) with curcumin. The insets represent the AUC values (g/dL/120 min). Values are expressed as means ± SEM, *n* = 7-8 per group. Differences between groups were analyzed with one-way ANOVA followed by Student-Newman-Keuls test (*P* < 0.05). #: differences with NYOG and NC90; ∗: differences with DYOG.

**Figure 4:**
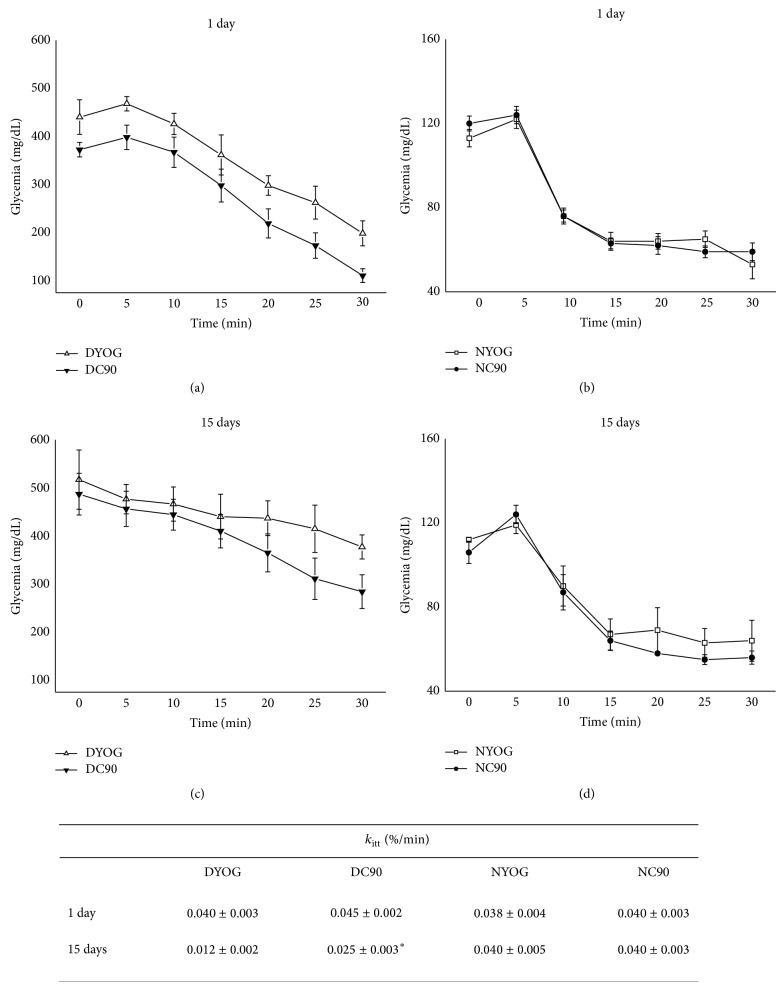
Insulin sensitivity of normal and STZ-diabetic rats treated with curcumin incorporated in yoghurt. Glycemia levels after insulin administration in rats treated for 1 day ((a) diabetic; (b) normal) and for 15 days ((c) diabetic; (d) normal) with curcumin. The inserted table shows the *k*_itt_ values (%/min), which were calculated as percentage of the respective control group (DYOG or NYOG). Values are expressed as means ± SEM, *n* = 7-8 per group. Differences between groups were analyzed with one-way ANOVA followed by Student-Newman-Keuls test (*P* < 0.05). ∗: differences with DYOG.

In the OGTT, the hyperglycemia peek developed by DYOG group 30 minutes after the glucose overload was approximately 2-fold higher (*P* < 0.001) than NYOG, in the 1st (Figures 3(a) and 3(b)) or 15th day (Figures 3(c) and 3(d)) of experiment. After 120 minutes, DYOG did not correct the glycemia with the same efficiency of that observed in NYOG, this last group returning the glycemia to basal values, in the 1st or 15th day of experiment. Consequently, the glucose tolerance was significantly impaired in DYOG rats when compared with NYOG rats (Figure 3, AUC, 1 and 15 days). Insulin sensitivity was progressively decreased in diabetic rats, since after insulin administration the DYOG group was not able to reduce the glycemia levels as efficiently as NYOG, in both 1st day (Figures 4(a) and 4(b)) and 15th day (Figures 4(c) and 4(d)) of experiment.

The treatment of normal, nondiabetic rats with curcumin (NC90) did not change the glucose tolerance in comparison to normal rats treated only with yoghurt (NYOG), after 1 (Figure 3(b)) or 15 days (Figure 3(d)) of treatment (Figure 3). The ITT was also performed in normal rats after 1 and 15 days of curcumin treatment, and the constant rate for glucose disappearance (*k*_itt_) was calculated to estimate the insulin sensitivity. There were no statistical differences in the *k*_itt_ values between NYOG and NC90 rats after 1 or 15 days of treatment (Figure 4, inserted table, N groups).

Although DYOG and DC90 groups showed similar values of hyperglycemia peek after the glucose overload, diabetic rats treated with curcumin for 1 day showed a better capacity to reduce the glycemia after 105 and 120 minutes of the glucose overload (*P* < 0.05, Figure 3(a)); however the glucose tolerance was not yet different when DYOG and DC90 were compared (Figure 3, AUC, 1 day). The insulin sensitivity was not also different for the comparison between DYOG and DC90 after 1 day of treatment, since these groups showed similar rates of glucose disappearance after insulin administration and similar *k*_itt_ values (Figure 4, inserted table, D groups).

After 15 days of treatment, the beneficial effects of curcumin on glucose metabolism of diabetic rats become evident. In the OGTT, after 60 minutes of the glucose overload, DC90 rats were able to reduce glycemia more efficiently than DYOG (Figure 3(c)); consequently, the glucose tolerance was significantly improved in DC90 group in comparison with DYOG (*P* < 0.05, Figure 3, AUC, 15 days). The treatment of diabetic rats with curcumin for 15 days reduced the fall in the insulin sensitivity, and the *k*_itt_ values of DC90 rats were 2-fold increased (*P* < 0.01) when compared with DYOG (Figure 4, inserted table, D groups).

### 3.4. AKT Phosphorylation Levels and GLUT4 Content in Muscle Plasma Membrane after Long-Term Treatment of Diabetic Rats with Curcumin

**Figure 5:**
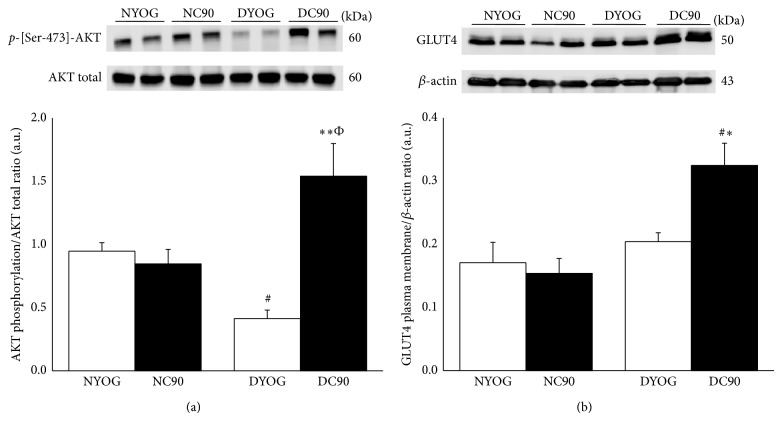
AKT activation (a) and plasma membrane GLUT4 content (b) in* gastrocnemius* muscles of normal and STZ-diabetic rats treated with curcumin incorporated in yoghurt for 15 days. AKT activation was evaluated as phosphorylation levels in Ser-473 residues. Results are expressed as means ± SEM of the arbitrary units, *n* = 5-6 per group. Differences between groups were analyzed with one-way ANOVA followed by Student-Newman-Keuls test. # (*P* < 0.05): differences with NYOG and NC90; ∗ (*P* < 0.05) and ∗∗ (*P* < 0.01): differences with DYOG; Φ (*P* < 0.05): differences between DC90 and NC90.

Phosphorylation levels of AKT were significantly lower (approximately 50%, *P* < 0.05) in* gastrocnemius* muscles of diabetic rats treated with yoghurt (DYOG) when compared with NYOG or NC90 rats. There were no differences in the AKT phosphorylation levels between NYOG and NC90 rats (Figure 5(a)). On the other hand, after the glucose overload, the phosphorylation of AKT was significantly increased in* gastrocnemius* of diabetic rats treated with curcumin when compared with DYOG (3.7-fold, *P* < 0.01) and with NC90 (82%, *P* < 0.05) rats (Figure 5(a)).

After a glucose overload, the levels of GLUT4 in plasma membrane of NYOG, NC90, and DYOG rats were very similar (Figure 5(b)). However, GLUT4 content was increased in plasma membrane of* gastrocnemius* muscles of diabetic rats treated with curcumin (DC90) when compared with DYOG rats (59%, *P* < 0.05) and with NYOG and NC90 rats (45 and 38%, resp., *P* < 0.05) (Figure 5(b)).

## 4. Discussion

The values of *C*_max⁡_ and *t*_max⁡_ found in the present study after the oral administration of 500 mg/kg curcumin were similar to those of the study by Yang et al. [23], which administered to rats the same curcumin dose, although they did not mention the vehicle. The curcumin half-life in our study was 32.70 ± 12.92 minutes (Table 4), while Yang and colleagues found it to be 44.5 ± 7.5 minutes [23]. Although the dose and the route of administration were similar between the studies, it can be suggested that the slight difference in the half-life values may be due to differences in the animal's body weight, since Yang and colleagues used rats weighing between 280 and 320 g, which could be influencing the *V*_*d*_ and increasing the elimination half-life of curcumin.

There are several studies investigating the curcumin pharmacokinetic parameters; however there were some differences between them, such as the vehicle used for administration, dose, and animal body weight, which make the comparison difficult as can be seen in Table 3; these studies indicate clearly that levels of curcumin are not directly comparable. The *C*_max⁡_ and AUC values obtained in the oral administration of curcumin were significantly lower than those found in the i.v. administration, and this fact suggests a low absorption and/or an increased hepatic first-pass metabolic biotransformation when curcumin is administered orally. The oral bioavailability of curcumin in rats was 0.47 ± 0.12% (Table 4).

Several curcumin pharmacokinetic studies in the last decade showed low absorption and fast metabolism, leading to a decrease of its bioavailability. Thereby, curcumin plasma levels are extremely low; however it is highly efficient as a therapeutic agent. Therefore, it should be reasonable to assume that some of its biological activities are associated with curcumin metabolite(s). In addition, it has been shown that, after its absorption, curcumin undergoes conjugation to glucuronide or sulfate derivatives [24]. Zhongfa et al. [25] demonstrated that the major curcumin metabolites are curcumin-o-glucuronide, tetrahydrocurcumin (THC), and curcumin-o-sulfate. The curcumin absorption in rats occurs through intestinal cells and most of it is eliminated as curcumin glucuronide or sulfate [26].

Evidences about the biological activity of curcumin metabolites are increasing. THC is one of the most studied, which showed bioavailability higher than curcumin [27]. Studies have shown that THC has some biological activities, such as anti-inflammatory [28], hepatoprotective [29], antidiabetic [27, 30], and antioxidant [31] activities; the most are higher when compared to curcumin effects.

The curcumin half-life was 32.70 ± 12.92 minutes (Table 4); that is, no curcumin should be bioavailable after 5.45 hours, because of the wash-out in ten half-lives [32]. Thus, it is possible to assume that one or more curcumin metabolites are responsible for its antidiabetic activity, since glycemia reduction in STZ-diabetic rats treated with curcumin incorporated in yogurt at intervals of 12 hours was observed [14].

Based on the aforementioned half-life considerations, the evaluation of changes in the glucose tolerance and insulin sensitivity of normal and diabetic rats after acute (1 day) and subchronic (15 days) treatments with curcumin-enriched yoghurt was performed. For this, we considered that the administration of glucose (OGTT) or insulin (ITT) would be carried out two hours after the curcumin administration, which means the elapsed time of approximately 4 half-lives; at this time, the curcumin concentration should be in critical levels and so its metabolites would be present and at pharmacological levels [14]. Therefore, we could investigate the pharmacodynamic effects on glycemia at intervals of 2–4 hours and 2–2.5 hours for OGTT and ITT, respectively.

STZ administration to rats promotes pancreas beta cell destruction and decreases the insulin secretion; therefore, the hyperglycemia in STZ-diabetic rats appears within 12–48 hours after STZ injection [33]. Thus, the glucose intolerance of STZ-diabetic rats may be explained, at least in part, by their deficiency in secrete insulin after a glucose overload. Very low plasma insulin levels in STZ-diabetic rats after a glucose overload have been observed [34], which could explain our data related to the impaired glucose tolerance in rats with 4 and 19 days of diabetes, related to experiments carried out for 1 and 15 days, respectively (Figures 3(a) and 3(c)).

Insulin resistance is defined as an attenuated biological response of tissues to physiological or elevated levels of insulin. According to our data, STZ-diabetic rats showed a diminished rate of glucose disappearance after insulin administration, in the 1st day of the experiment (4 days after STZ, Figure 4(a)), which was even impaired in the 15th day (19 days after STZ, Figure 4(c)). Evidences support the insulin resistance observed in STZ-diabetic animals, mainly characterized by a reduction in the tyrosine kinase activity of the insulin receptor (IR), despite the increased IR number [35, 36] and decrease in the AKT activation (phosphorylation in serine-473 residue) [37]. According to our data, diabetic rats after 19 days of the STZ administration and treated with yoghurt showed a profile compatible with insulin resistance, since the muscle AKT phosphorylation was lower than values of normal rats (Figure 5(a)). In addition to the direct disturbances in the insulin signaling of STZ-diabetic rats, the worsening in the insulin responsiveness observed with the progression of the diabetes may be also related to changes in the content of glucose transporters in skeletal muscles. Kahn et al. [38] observed that the levels of glucose transporters types 1 and 4 (GLUT1 and GLUT4) are progressively decreased in muscles of STZ-diabetic rats with the extent of the diabetes, although in this present study a reduction in GLUT4 content in muscle plasma membrane of diabetic rats (DYOG) in comparison with normal rats (NYOG) was not found, after a glucose overload (Figure 5(b)); these differences may be due to periods of diabetes studied (Kahn study: 7–14 days; present study: 19 days).

The similarity in the rate of glucose disappearance after a glucose overload (Figures 3(b) and 3(d)) or insulin administration as well as in the *k*_itt_ values (Figures 4(b) and 4(d) and inserted table) of normal rats after 1 and 15 days of treatment represents that the tissue responsiveness to insulin is maintained in these animals over the experimental period. Both pancreas insulin secretion and peripheral insulin responsiveness are well-functioning in the correction of hyperglycemia states, so it is reasonable to understand the absence of effects of curcumin in the glucose tolerance and in the insulin sensitivity of nondiabetic rats. Also, AKT phosphorylation and GLUT4 plasma membrane levels are also similar between NYOG and NC90 rats (Figures 5(a) and 5(b)). These findings corroborate previous data from our laboratory, showing that even a chronic daily treatment (31 days) with curcumin-enriched yoghurt did not change the postprandial glycemia levels of normal rats [14].

Our results showed that one single administration of curcumin was not sufficient to improve the ability of STZ-diabetic rats to reverse hyperglycemia. However, in the 1st day of curcumin treatment, it is interesting to note that these rats showed a fast decrease in the glycemia after 105 and 120 minutes of the glucose overload, when compared with untreated-diabetic rats (Figure 3(a)). This finding reiterates the pharmacokinetic data on the possibility of the beneficial effects of curcumin to be exerting by a metabolite(s), which need an additional time to reach the therapeutic levels and to control the glucose metabolism. In fact, it was observed that the treatment for 15 days with curcumin promoted a most evident benefit on glucose metabolism of diabetic rats, improving both the glucose tolerance (Figure 3(c)) and the insulin sensitivity (Figure 4(c) and inserted table). Long-term of a daily treatment with curcumin probably culminates in an increased permanence into the circulation of the curcumin metabolite(s), allowing the biological active compound(s) to exert its effects in a most pronounced way. Recent evidence of our laboratory reinforces the possibility of the antidiabetic activity of curcumin to be exerted by metabolite(s): Arcaro et al. [15] found that the treatment of STZ-diabetic rats with yoghurt enriched with 90 mg/kg curcumin and piperine did not increase (20 mg/kg piperine) and even nullified (40 mg/kg piperine) the antidiabetic and antioxidant activities of curcumin. It is well known that piperine increases the bioavailability of many drugs and compounds, including curcumin, via inhibition of the activity of various metabolizing enzymes found in liver and intestine, such as aryl hydroxylases, N-demethylases, UDP-glucuronyltransferases, and cytochrome P450 3A4 [39, 40]. However, the inhibition of the curcumin biotransformation will not necessarily lead to an increase of its pharmacodynamic actions, and indeed adverse effects can be reached.

Corroborating the hypothesis that curcumin metabolite(s) has pivotal importance in determined biological activities when curcumin is administered orally, Neyrinck et al. [41] found that the coadministration of curcuma extract (0.1% of curcumin) and 0.01% of white pepper (which contains piperine) to mice fed a high-fat (HF) diet did not promote any change in the glucose and lipid homeostasis, in comparison with nontreated HF mice. Besides, HF mice receiving these phytotherapics showed low levels of proinflammatory cytokines IL-6 and TNF-*α* in subcutaneous adipose tissue in association with accumulation of THC; the authors suggested that this metabolite may be responsible for the anti-inflammatory response.

The findings of the present study showed that diabetic rats treated for 15 days with curcumin incorporated in yoghurt had an increase in the AKT phosphorylation in* gastrocnemius* muscles (Figure 5(a)) in response to a glucose overload, which may explain the increased GLUT4 content (Figure 5(b)) in muscle plasma membranes of these rats, in comparison with diabetic rats treated only with yoghurt. Taken together, these results suggest that curcumin-enriched yoghurt increased the glucose uptake by skeletal muscles, which may account for the fast decrease in the hyperglycemia in DC90 rats after a glucose overload. Considering that the total GLUT4 content was not changed in muscles of diabetic rats by curcumin treatment (data not shown), it can be suggested that curcumin increased the GLUT4 translocation to plasma membrane, probably due to a direct AKT activation and/or by increase in the tissue responsiveness to insulin and/or by stimulating the pancreas insulin release. Although there are studies showing the ability of curcumin to promote AKT activation [42] and to increase the GLUT4 content in plasma membranes of skeletal muscles cells [43, 44], the possibility of these responses to be exerted by curcumin metabolites cannot be ruled out, which is corroborated by the effect of the coadministration of piperine (40 mg/kg) and curcumin (90 mg/kg) [15]. In addition, Murugan and collaborators [45] showed that erythrocytes from STZ-nicotinamide diabetic rats treated for 45 days with curcumin (80 mg/kg) or with THC (80 mg/kg) showed an increased ability for insulin-receptor binding when compared with cells from nontreated diabetic rats, which was associated with the antihyperglycemic effect of these compounds.

## 5. Conclusion

The present findings in the half-life of curcumin in plasma of diabetic rats (PK) and in the temporal behavior in the glucose tolerance and insulin sensitivity assays after acute and subchronic treatments with curcumin-enriched yoghurt (PD) are inconsistent, which substantiate evidences for curcumin metabolite(s) as being responsible for the antidiabetic activity which may be related, at least in part, to an increase in skeletal muscle glucose uptake due to AKT activation leading to an enhancement in the plasma membrane GLUT4 content.

## Figures and Tables

**Table 1:** Intraday (*n* = 5) and interday (*n* = 5) accuracy and precision of the bioanalytical method to quantify curcumin in plasma.

Nominal concentration (ng/mL)	Accuracy (%)	Precision CV (%)
Intraday		
10	113.59	13.52
25	86.05	11.03
125	92.40	2.67
400	112.88	8.68
Interday		
10	89.65	7.58
25	91.85	12.46
125	94.52	8.57
400	98.74	9.62

**Table 2:** Stability of curcumin in plasma under different conditions of storage, temperature, and time intervals (*n* = 3).

Sample condition	Curcumin nominal concentration			
	25 ng/mL		400 ng/mL	
	Accuracy (%)	Precision CV (%)	Accuracy (%)	Precision CV (%)
*Bench-top* (30°C, 4 h)	91.85	8.90	105.90	12.50
*Postprocessing* (30°C, 4 h)	88.45	7.50	101.56	13.40
*Freeze-thaw* (−20°C, 72 h)	89.34	5.40	99.47	11.60
*Long time* (−20°C, 7 d)	90.77	6.20	103.24	8.90

**Table 3:** Rat plasma/serum levels of curcumin administered in different vehicles.

Animal	Administration	Curcumin (g/kg)	Vehicle	*C*_max⁡_ (*μ*g/mL)	*t*_max⁡_ (hour)
Diabetic rat^#^	Oral	0.50	Curcumin-enriched yoghurt (0.5 mL)	0.060	0.25
Diabetic rat^#^	Intravenous	0.01	Curcumin in DMSO (0.5 mL/kg)	3.140	0.08
Normal rat [46]	Oral	0.10	Curcumin powder	0.084	2.00
Normal rat [46]	Oral	0.10	Marketed CUR-500 capsules	0.092	2.00
Normal rat [46]	Oral	0.10	Curcumin nanocrystal-loaded capsules	0.041	0.50
Normal rat [47]	Oral	0.05	Curcumin aqueous suspension	0.004	0.50
Normal rat [47]	Oral	0.05	Curcumin-loaded PLGA nanoparticles	0.011	2.00
Normal rat [47]	Oral	0.05	Curcumin-loaded PLGA-PEG blend nanoparticles	0.029	3.00
Normal rat [48]	Oral	0.10	Curcumin nanoparticles	0.260	2.00
Normal rat [48]	Oral	0.25	Curcumin + piperine (0.01 g/kg) water suspension	0.121	0.75
Normal rat [48]	Oral	0.25	Curcumin suspension	0.090	0.50
Normal rat [23]	Oral	0.10	Curcumin liposome dissolved in saline	0.042	0.30
Normal rat [49]	Oral	0.05	Curcumin solubilized in Tween 80	292.000	0.25
Normal rat [49]	Oral	0.05	Curcumin loaded solid lipid nanoparticles	14.290	0.50
Normal rat [50]	Oral	0.50	∗	0.060	0.68
Normal rat [50]	Intravenous	0.01	∗	0.360	∗
Normal rat [51]	Oral	1.00	Curcumin phospholipid complex	1.200	1.50
Normal rat [51]	Oral	1.00	∗	0.500	0.75
Normal rat [52]	Oral	0.34	Curcumin phospholipid complex	0.012	0.25
Normal rat [52]	Oral	0.34	Curcumin unformulated	0.002	0.50
Normal rat [53]	Oral	0.30	Curcumin phospholipid complex	0.600	2.33
Normal rat [53]	Oral	0.10	∗	0.266	1.62
Normal rat [12]	Oral	2.00	Curcumin + piperine (0.02 g/kg) water suspension	1.800	1.29
Normal rat [12]	Oral	2.00	Curcumin aqueous suspension	1.350	0.83

^#^This study.

^∗^Not reported. *C*_max⁡_: peak plasma/serum concentration; *t*_max⁡_: time to reach peak plasma/serum concentration.

**Table 4:** Pharmacokinetic parameters after oral (500 mg/kg) and i.v. administration (10 mg/kg) of curcumin to STZ-diabetic rats. Values are expressed as means ± SEM (*n* = 5).

Pharmacokinetic parameters	Administration route	
	Oral	i.v.
*k*_el_ (1/min)	0.02 ± 0.01	0.08 ± 0.02
Half-life (min)	32.70 ± 12.92	8.64 ± 2.31
AUC_0-*t*_ (*μ*g/mL/min)	1.89 ± 0.25	12.27 ± 2.75
AUC_0-*∞*_ (*μ*g/mL/min)	2.97 ± 0.79	12.45 ± 2.72
Cl (L/kg/min)	0.85 ± 0.24	0.83 ± 0.19
*V*_*d*_ (L/kg)	37.49 ± 10.46	10.63 ± 4.10
MRT (min)	55.41 ± 20.19	12.46 ± 3.34
*k*_*a*_ (1/min)	0.29 ± 0.15	—
*C*_max⁡_ (*μ*g/mL)	0.06 ± 0.01	3.14 ± 0.90
*t*_max⁡_ (min)	15.00 ± 0.00	5.00 ± 0.00
*F* (%)	0.47 ± 0.12	100

*k*_el_: elimination constant; half-life: time half-life; AUC_0-*t*_: area under plasma concentration/time plot until the last quantifiable value; AUC_0-*∞*_: area under plasma concentration/time plot extrapolated to infinity; Cl: clearance; *V*_*d*_: volume of distribution; MRT: average mean residence time; *k*_*a*_: absorption constant; *C*_max⁡_: maximum concentration; *t*_max⁡_: time to reach *C*_max⁡_; *F*: bioavailability.
